# Application of noninvasive prenatal testing-plus in fetal ultrasound cardiovascular abnormalities: An observational study

**DOI:** 10.1097/MD.0000000000046108

**Published:** 2025-11-28

**Authors:** Keqin Jin, Xiayuan Xu, Shuangshuang Shen, Min Hu, Jianfeng Luo, Na Li

**Affiliations:** a Jinhua’s Key Laboratory of Birth Defects Prevention and Treatment, Jinhua, Zhejiang, China; b Genetic Laboratory, Jinhua Maternal & Child Health Care Hospital, Jinhua, Zhejiang, China; c Gynaecology and Obstetrics, Jinhua Maternal & Child Health Care Hospital, Jinhua, Zhejiang, China; d Teaching Affairs Office, Affiliated Jinhua Hospital of Zhejiang University School of Medicine, Jinhua, Zhejiang, China.

**Keywords:** congenital heart disease, copy number variation, NIPT-Plus, prenatal diagnosis

## Abstract

Congenital heart disease (CHD), the most common birth defect in China with high neonatal mortality, is frequently associated with chromosomal abnormalities including copy number variations (CNVs), yet conventional ultrasound screening has limitations in detecting these genetic underpinnings; noninvasive prenatal testing plus (NIPT Plus) may address this gap, but its clinical efficacy in diagnosing fetal CHD remains unclear. To explore the clinical efficacy of NIPT Plus in the diagnosis of fetal CHD. Pregnant women whose fetuses were identified with congenital structural heart defects or abnormal cardiac soft markers via ultrasound between August 2019 and September 2023 were enrolled. All participants underwent NIPT-Plus and invasive prenatal diagnostic procedures, including chromosome karyotyping and/or chromosomal microarray analysis (CMA). The concordance between CNVs detected by NIPT-Plus and results from invasive prenatal diagnosis was analyzed. Among fetuses with congenital structural cardiac malformations or abnormal cardiac soft markers identified by prenatal ultrasound, 39 cases underwent NIPT-Plus testing, with a positive rate of 30.77% (12/39). Seven cases withdrew from the study for personal reasons after NIPT-Plus showed no abnormalities, and the remaining 32 cases received further confirmation via invasive prenatal diagnosis. Of these 32 cases, 12 were positive for NIPT-Plus, 11 of which were consistent with CMA validation results, yielding a positive predictive value of 91.67%. Additionally, 10 cases had CNVs > 4 Mb, 9 of which matched CMA results, with a positive predictive value of 90%. No significant difference was observed between NIPT-Plus and CMA results. NIPT-Plus exhibits favorable detection performance and clinical utility in the auxiliary diagnosis of fetal cardiovascular developmental abnormalities. However, in clinical practice, its results should be validated in combination with ultrasound findings and invasive tests such as amniocentesis to minimize the risks of false positives and missed diagnoses.

## 1. Introduction

Congenital heart disease (CHD) ranks 1st among all birth defects, with approximately 130,000 to 180,000 new cases reported annually in China, accounting for roughly 1% of all newborns. Alarmingly, 60% of affected infants succumb to the condition within their 1st year of life.^[1]^ CHD frequently co-occurs with hereditary disorders or forms part of specific syndromic conditions. It is estimated that 15% to 30% of children with CHD have underlying chromosomal abnormalities, including aneuploidies, microdeletions, microduplications, and monogenic disorders,^[2,3]^ with copy number variations (CNVs) contributing to approximately 10% of CHD cases.^[4,5]^

Currently, ultrasonography remains the primary method for prenatal screening of CHD; however, its performance is heavily influenced by factors such as the operator’s expertise and gestational age.^[6]^ A meta-analysis by Junshu et al^[7]^ revealed that even with well-equipped facilities and experienced sonographers, the prenatal ultrasound detection rate for severe congenital anomalies ranges from 40% to 70%, with significantly lower rates for rare anomalies. This underscores an urgent need to enhance the detection efficiency of fetal CHD, thereby offering more informed options to affected families.^[8]^

In 1997, Lo et al^[9]^ pioneered the application of cell-free fetal DNA in noninvasive prenatal diagnosis, successfully isolating fetal-derived free DNA from maternal peripheral blood, where maternal DNA predominates. Early iterations of this technology, however, were constrained by stringent requirements for instrumentation and technical proficiency,^[10,11]^ which limited its utility to accurate screening for major chromosomal aneuploidies, while detecting smaller CNVs remained challenging. Consequently, identifying CHD caused by microdeletions or microduplications demanded more sensitive and precise noninvasive prenatal testing (NIPT) methodologies.

In recent years, noninvasive prenatal testing plus (NIPT-Plus) has emerged as a breakthrough in prenatal screening for CHD, potentially addressing this diagnostic gap. However, its efficacy in detecting small-sized CNVs (particularly those closely linked to congenital heart disease) remains insufficiently characterized. Thus we aimed to explore the clinical performance of NIPT-Plus in fetuses with ultrasound-identified CHD.

## 2. Materials and methods

### 2.1. Ethics statement

The study was approved by the Ethics Committee of Jinhua Maternal & Child Health Care Hospital (No.: 2019-KY-016). All procedures implemented within this study were strictly in accordance with the ethical guidelines formulated by the ethics committee of Jinhua Maternal & Child Health Care Hospital. Written informed consent was duly obtained from every study participant. Moreover, all methods were carried out in full compliance with relevant guidelines and regulations, ensuring the integrity and ethical soundness of the entire research process.

### 2.2. Information

**Figure 1.**
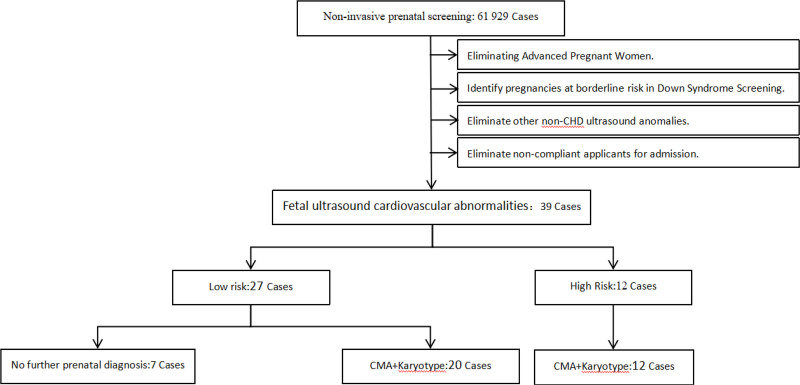
Prenatal genetic testing among 39 fetal ultrasound cardiovascular abnormalities cases.

A total of 61,929 pregnant women underwent noninvasive prenatal screening at Jinhua Maternal & Child Health Care Hospital and the Maternal and Child Health Hospitals of the subordinate county-level cities in Zhejiang Province from August 2019 to September 2023. Among them, 39 pregnant women met the inclusion criteria and voluntarily signed the informed consent for NIPT Plus testing. Prenatal diagnosis was carried out for 32 of these 39 women (refer to Fig. 1). Inclusion criteria: pregnant women with a singleton pregnancy, aged 17 to 44 years, who signed the informed consent. Pregnant women in whom prenatal ultrasound examination detected congenital cardiac structural malformations or abnormalities in soft cardiac ultrasound indexes.

### 2.3. NIPT-Plus

After each participant received the necessary information, they signed an informed consent form. Subsequently, 10 mL of venous blood was extracted. The plasma was removed, and a DNA extraction kit (Yue Dong Meibei 20170019, Dongguan Boao Muhua Gene Technology Co. Ltd.) was used to extract DNA from the remaining sample, free of plasma.

Prior to March 2022, each sample underwent processing with the Jingxin Fetal Chromosome Aneuploidy (T21, T18, T13) Detection Kit (Semiconductor Method; National Approval Certificate: 20153400300; Boao Biological Group Co., Ltd.) for the preparation of sequencing templates and library construction. High-throughput sequencing was subsequently carried out using the BioelectronSeq 4000 (Boao Biological Group Co., Ltd.). Commencing in March 2022, Berry Medical Laboratories, Inc. undertook analysis of all samples. The samples were then sequenced on the Illumina NextSeq CN500 platform using the RUPA data analysis system.

After sequencing quality control procedures, the BWA software was deployed to align reads, locate, and extract unique reads. In accordance with the study by Liao C et al,^[12]^ the obtained data were standardized and corrected. This enabled the prediction of fetal aneuploidies and CNVs. To predict fetal chromosome aneuploidies, a general function was utilized to calculate the *Z*-value. For the determination of fetal CNVs, Stouffer *Z*-value was computed following the approach described by Yin AH et al^[13]^ CNVs were reported when their size was equal to or >0.5 Mb.

### 2.4. Karyotype analysis

Fully informed prenatal diagnosis and signed informed consent. Under the guidance of ultrasound, amniotic fluid was withdrawn for inoculation, harvesting, slicing, and G-banding according to in situ or digestive methods, and karyotyping was performed according to ISCN (2016) standards.

### 2.5. Chromosomal microarray analysis (CMA)

Amniotic fluid samples were subjected to CMA using the Affymetrix 750K microarray kit [CytoScan 750K Array Kit and Reagent Kit Bundle (Catalog No. 901859)]. The analysis followed standard laboratory procedures recommended by the kit manufacturer. Resultant data were analyzed with ChAS v.3.0 software. The entire analysis process adhered strictly to the guidelines of the American College of Medical Genetics and Genomics (ACMG).

Subsequently, the genomes of fetuses with verified CHD were screened for DNA CNVs. To ensure consistency in reporting, the threshold for CNV size was set at ≥ 0.5 Mb. This threshold was applied uniformly across all CNV detection efforts, maintaining methodological integrity throughout the study.

### 2.6. Ultrasonography

Pregnant women were examined by systematic ultrasound using a GE Voluson 730 and/or GE Voluson E10 (Wuxi, China) ultrasound Doppler device (probe frequency 2.5–5.0 MHz).

### 2.7. Recall and follow-up of pregnant women

Pregnant women screened positive for the new NIPT were recalled and underwent prenatal diagnosis and ultrasound diagnosis, while NIPT-negative pregnant women were followed up for fetal status by telephone and routine physical examination.

### 2.8. Statistical processing

SPSS 20.0 software (IBM Corporation, Armonk) was used for statistical analysis, and the count data were expressed as rates, and the *χ*^2^ test was used for comparison between groups, with *P *< .05 as the difference being statistically significant.

## 3. Results

### 3.1. Analysis of the clinical efficacy of NIPT Plus in ultrasound-indicated CHD fetuses

**Figure 2.**
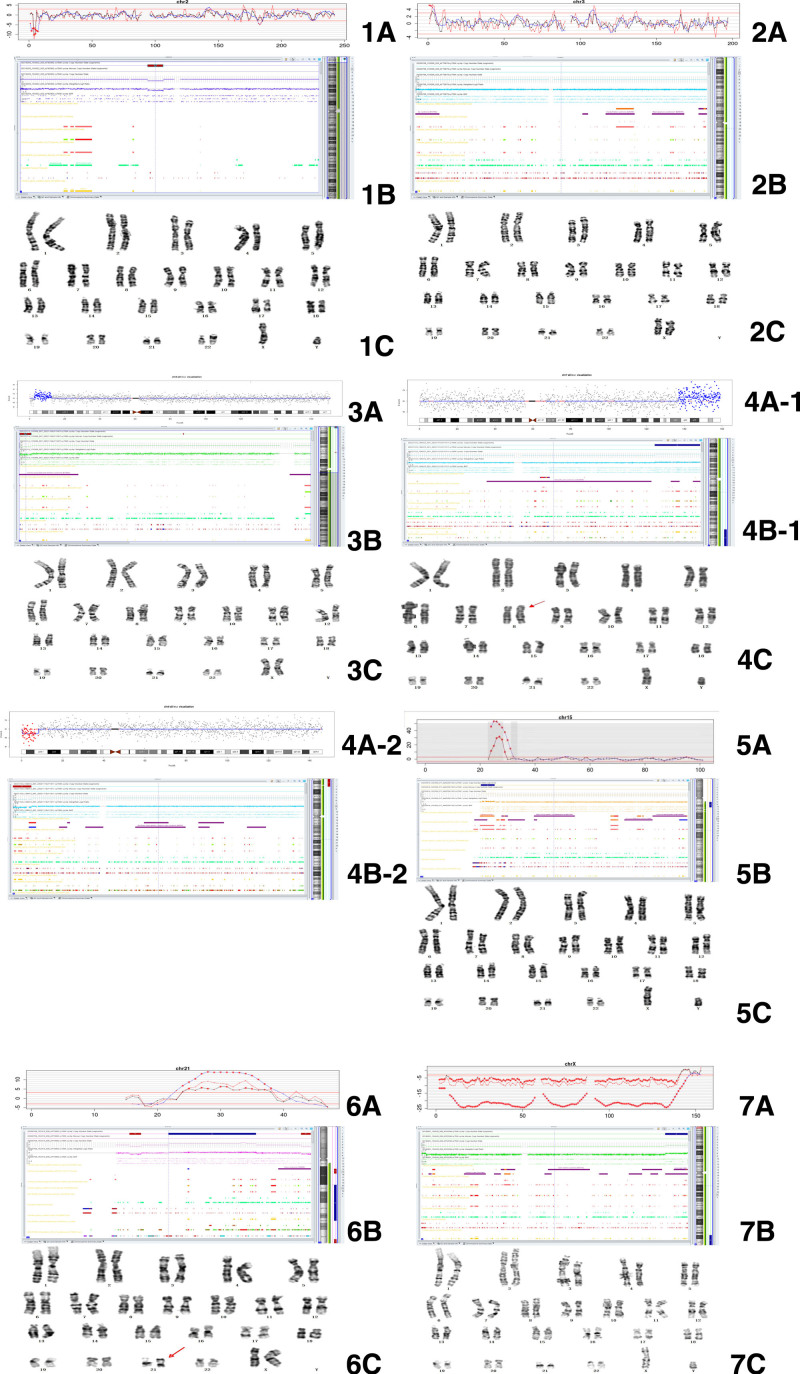
The verification result figure of chromosome microarray analysis (CMA) combined with karyotype for the copy number variations (CNVs) indicated by NIPT Plus in 7cases:①Indicated by NIPT Del(2)(p25.3-p25.2)(4.12M-4.62M): 1A chr2 all in z visualization, 1B CMA detailed diagram of the deletion on chromosome 2, 1C chromosome karyotype diagram; ②Indicated by NIPT Dup(3)(p26.3-p26.1)(0–4M): 2A chr3 all in z visualization, 2B CMA detailed diagram of the duplication on chromosome 3, 2C chromosome karyotype diagram;③Indicated by NIPT Del(6)(p25.3-p25.2)(0.5M-2.5M): 3A chr6 all in z visualization, 3B CMA detailed diagram of the deletion on chromosome 6, 3C chromosome karyotype diagram; ④ Indicated by NIPT Dup(7)(q32q36.3)(126M-159M), Del(8)(p23.3p23.1)(0.2M-8.2M): 4A-1 chr7 all in z visualization, 4A-2 chr8 all in z visualization,4B-1 CMA detailed diagram of the duplication on chromosome 7, 4B-2 CMA detailed diagram of the deletion on chromosome 8, 4C chromosome karyotype diagram;⑤Indicated by NIPT Dup(15)(q11.2-q13.1)(23.6M-28.56M): 5A chr15 all in z visualization, 5B CMA detailed diagram of the duplication on chromosome 15, 5C chromosome karyotype diagram;⑥Indicated by NIPT Dup(21)(q21.1-q22.12)(23M-37M): 6A chr21 all in z visualization, 6B CMA detailed diagram of the duplication on chromosome 21, 6C chromosome karyotype diagram;⑦Indicated by NIPT Dup(X)(q27.2-q28)(142M-153M): 7A chrX all in z visualization, 7B CMA detailed diagram of the duplication on chromosome X, 7C chromosome karyotype diagram.

During the study of 39 cases with fetal congenital cardiac malformations/abnormal soft cardiac ultrasound indicators detected by ultrasound during pregnancy, NIPT Plus was executed. Positive findings were noted in 12 cases, depicting a detection rate of 30.77%. Seven subjects withdrew from the investigation following negative results by NIPT Plus, while the remaining 32 cases underwent prenatal diagnostic verification. Out of these, 32 ultrasounds with structural cardiac anomalies or indicators were confirmed (Table 1). The verification results of the CNVs indicated by NIPT Plus in 7 cases, which were analyzed by combining CMA with karyotype analysis, are shown in detail in Figure 2. Among the 32 cases with cardiac anomalies or indicators, 12 were identified positively by NIPT Plus, with 11 matching the CMA validation results, indicating a positive compliance rate of 91.67%. Notably, 10 cases of CNVs > 4M were detected, with 9 matching the CMA validation results, signifying a positive compliance rate of 90% (Table 2).

**Table 1:** Comparison of aneuploidy and CNVs screened by NIPT Plus with chromosomal karyotype and CMA validation results.

NIPT Plus	Number of cases	Karyotype	Validation	Number of cases
T13	2	47,XY,+13	arr(13) × 3	2
T18	2	47,XX,+18	arr(18) × 3	2
T21	1	47,XX,+21 [50]/46,XX[2]	arr(21) × 3	1
Dup(X)(q27.2-q28)(142M-153M),12M	1	46,XY	arr[hg19] Xq27.2q28(142,095,573–155,233,098) × 2	1
Dup(3)(p26.3-p26.1)(0–4M),5M	1	46,XX	arr(1–22) × 2,(XN)x1	1
Dup(7)(q32q36.3)(126M-159M),34M; Del(8)(p23.3p23.1)(0.2M-8.2M),8M	1	46,XY,der(8)	arr[hg19] 7q33q36..3 (134,471,302–159,119,707) × 3,8p23.3p23..1 (158,049-9386,993) × 1	1
Dup(15)(q11.2-q13.1)(23.6M-28.56M),4.96Mb	1	46,XY	arr[hg19] 15q11.2q13..1 (23,286,423-28,526,905) × 3	1
Dup(21)(q21.1-q22.12)(23M-37M),15M	1	46,XX,der(21)(q21q22)	arr[hg19] 21q21..1 (17,442,316-19,408,302) × 1, 21q21.2q22..13 (24,041,668-38,855,202) × 3,21q22..3 (46,464,874-48,093,361) × 1	1
Del(2)(p25.3-p25.2)(4.12M-4.62M),0.5M	1	46,XY	arr[hg19] 2p25.3p25..2 (4131,413-4635,997) × 1	1
Del(6)(p25.3-p25.2)(0.5M-2.5M),2.00Mb	1	46,XX	arr[hg19] 6p25..3 (156,975-2793,238) × 1	1
No apparent abnormality	20	46,XN	arr(1–22) × 2,(XN)x1	18
		46,XX,inv(9)	arr(1–22) × 2,(XN)x1	1
		46,XX,13pstk+	arr[hg19] 7q11..21 (64,612,880–65,162,169) × 1	1

CMA = chromosomal microarray analysis, CNV = copy number variation, NIPT Plus = Noninvasive Prenatal Testing Plus.

**Table 2:** Results of NIPT Plus detection of CNVs or aneuploidy validation in ultrasound cardiac abnormalities.

Categories	Number of high-risk cases of NIPT plus	Number of cases diagnosed with G-band/CMA	Number of positive cases	PPVs (%)	Follow-up results
CNV (>4M)or aneuploidy (in chromosomes)	10	10	9	90%	9 terminations of pregnancy; 1 normal birth
CNV(≥0.5 M ≤ 4M)	2	2	2	100%	1 normal birth;1 refusal of information
Total	12	12	11	91.67%	

PPV = positive predictive value.

### 3.2. Comparative analysis of NIPT Plus and CMA

Based on the chromosomal molecular karyotype gold standard CMA, NIPT Plus has a sensitivity of 91.67% (11/12) and a specificity of 93.75% ((11 + 19)/32). The comparison of NIPT Plus with prenatal diagnosis (CMA) showed that there was no statistical difference between the 2 techniques.

## 4. Discussion

In this study, we applied an optimized Fetal Copy-number Analysis through Maternal Plasma Sequencing algorithm to conduct NIPT Plus sequencing on maternal plasma samples from 39 CHD cases. The aim was to evaluate the diagnostic efficacy of NIPT Plus in detecting chromosomal aberrations associated with CHD. Consistent with prior research, our findings reaffirm that the sensitivity of NIPT Plus in identifying CNVs correlates with the size of chromosomal aberrations. The assay demonstrated a sensitivity of 91.67% and a specificity of 93.75%, aligning with our previous report.^[14]^ A notable observation was that CNVs ≤ 4 Mb exhibited a higher positive predictive value than those > 4 Mb, though this finding warrants cautious interpretation due to the limited sample size (n = 39) and potential statistical bias. Comparative analysis with CMA revealed no significant difference, an outcome likely influenced by the constrained statistical power inherent to small sample sizes. These results suggest that the optimized noninvasive testing approach holds promise as an alternative screening modality for patients who decline invasive prenatal diagnosis yet require fetal CNV assessment.

The 5 cases of pathogenic microdeletion and microduplication syndromes detected in this study. A case of 6p25.3 deletion, associated with a full-mutation deficient single dose score of 3 in the FOXC1 gene, related to Autosomal Dominant Axenfeld-Rieger Syndrome Type 3 (RIEG3) [OMIM:602482]. This specific genetic variant is primarily linked to PDA, ASD, bicuspid aortic valve, Dandy–Walker malformation, sensorineural hearing loss, oligodontia, iris hypoplasia, glaucoma, excessive furrow separation between the eyes, flat midface, saddle nose, and others.^[15,16]^ A case had overlapping 7q33q36.3 duplication and 8p23.3p23.1 deletion regions. Specifically, the 7q33q36.3 duplication encompassed a region within the 7q36.3 ZRS (SHH cis-regulatory) repeat zone (LMBR1 intron 5), a region that is given a triple dose sensitivity score of 3 points. Research articles (PMID: 15917205, 28035386, 2792209, 18178630, 18417549, 19291772) suggest that similar duplications in this zone can result in association with limb malformation phenotypes, such as preaxial polydactyly, type II (PPD2)[MIM #174500] and Werner mesomelic syndrome [MIM #188770]. Moreover, the 8p23.3p23.1 deletion region partially overlaps with the 8p23.1 deletion syndrome area. Patients with this syndrome commonly display characteristics including congenital heart disease, congenital diaphragmatic hernia, delayed development, and hyperactivity. A case of duplication of region 15q11.2q13.1, which is associated with 15q11q13 microduplication syndrome disease, with clinical phenotypes including autism, mental retardation, epileptic seizures, and psychiatric disorders, and occasional structural abnormalities of either of the heart and great vessels.^[17]^ A case of 21q21.2q22.13 segmental duplication compounding 21q21.1 and 21q22.3 deletions covering the APP gene, which is associated with dominantly inherited early-onset Alzheimer disease. A case of Xq27.2q28 duplication was associated with Xq28 duplication syndrome with clinical manifestations such as delayed speech and language development, mental retardation, hypotonia, recurrent infections, seizures, and spasticity. These findings highlight NIPT Plus’ potential to improve the detection of clinically relevant CNVs. While useful for prenatal screening of CHD-associated CNVs, it should complement (not replace) definitive diagnostics. NIPT Plus-detected CNVs require confirmation via karyotyping and CMA to guide genetic counseling. Larger studies are needed to clarify its utility across CNV sizes and establish interpretation guidelines.

## 5. Conclusions

NIPT-Plus exhibits robust performance in the auxiliary diagnosis of fetal cardiovascular malformations, with substantial clinical utility. However, in clinical practice, its results should be validated by invasive procedures such as amniocentesis to mitigate the risks of false positives and missed diagnoses. With the continuous advancement of next-generation sequencing technologies, NIPT-Plus is expected to achieve accurate detection of smaller CNV fragments, potentially evolving into a 1st-line screening tool for prenatal CNV assessment in clinical practice.

## Acknowldgments

We thank all patients who participated in this study.

## Author contributions

**Conceptualization:** Keqin Jin, Jianfeng Luo.

**Data curation:** Xiayuan Xu, Na Li.

**Funding acquisition:** Xiayuan Xu, Shuangshuang Shen, Min Hu, Jianfeng Luo, Na Li.

**Writing – original draft:** Keqin Jin.

**Writing – review & editing:** Keqin Jin, Min Hu.
